# Recent Advances in the Production of Pharmaceuticals Using Selective Laser Sintering

**DOI:** 10.3390/biomimetics8040330

**Published:** 2023-07-27

**Authors:** Athinarayanan Balasankar, Kandasamy Anbazhakan, Velusamy Arul, Velankadu Natrayan Mutharaian, Ganesan Sriram, Kanakaraj Aruchamy, Tae Hwan Oh, Subramaniyan Ramasundaram

**Affiliations:** 1 Department of Physics, Gobi Arts & Science College, Erode, Gobichettipalayam 638453, India; balphd1987@gmail.com (A.B.); aganphysics@gmail.com (K.A.); 2 Department of Chemistry, Sri Eshwar College of Engineering (Autonomous), Coimbatore 641202, India; arul.v@sece.ac.in; 3 Department of Botany, Gobi Arts & Science College, Erode, Gobichettipalayam 638453, India; mutharaian@gascgobi.ac.in; 4 School of Chemical Engineering, Yeungnam University, Gyeongsan 38541, Republic of Korea; sriram2085@gmail.com

**Keywords:** selective laser sintering, 3D printing, biomedicines, pharmaceuticals, drug release

## Abstract

Selective laser sintering (SLS) is an additive manufacturing process that has shown promise in the production of medical devices, including hip cups, knee trays, dental crowns, and hearing aids. SLS-based 3D-printed dosage forms have the potential to revolutionise the production of personalised drugs. The ability to manipulate the porosity of printed materials is a particularly exciting aspect of SLS. Porous tablet formulations produced by SLS can disintegrate orally within seconds, which is challenging to achieve with traditional methods. SLS also enables the creation of amorphous solid dispersions in a single step, rather than the multi-step process required with conventional methods. This review provides an overview of 3D printing, describes the operating mechanism and necessary materials for SLS, and highlights recent advances in SLS for biomedical and pharmaceutical applications. Furthermore, an in-depth comparison and contrast of various 3D printing technologies for their effectiveness in tissue engineering applications is also presented in this review.

## 1. Introduction

Manufacturing is often connected with the design and transformation of raw resources into consumable goods. The original method of production is subtractive manufacturing, in which the manufacturing process begins with raw materials and ends with the completed product. “Forming” is a type of manufacturing in which force is used to change a block of material into the product that is wanted. “Casting” is also a type of manufacturing where the solid substance is melted into a liquid and then poured into a mould, and the product is recovered upon cooling the mould [1]. The other type of manufacturing is called additive manufacturing (AM), in which parts are made by putting them together layer by layer. AM is the collective term for a wide variety of manufacturing processes used to create three-dimensional objects and prototypes from digital data. The basis for additive manufacturing is computer-aided design (CAD) [2,3,4]. Using data from solid modelling, additive models could make layers with very small cross-sections. This made it possible to make detailed and complicated structures and surfaces that would be hard to make with traditional methods [5].

**Figure 1:**
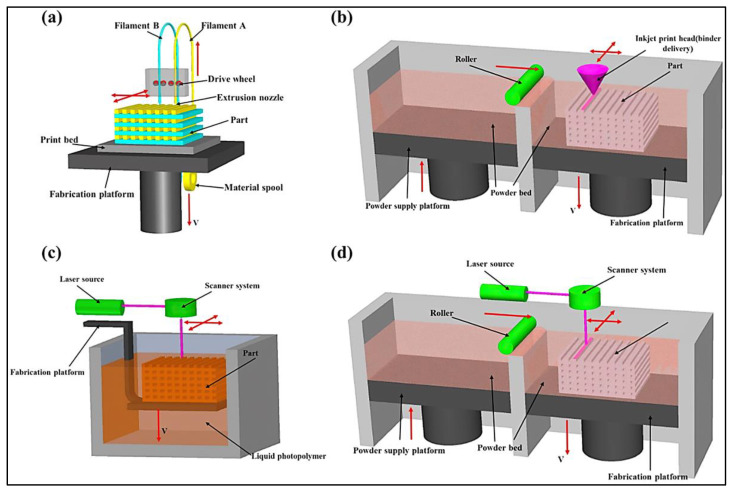
Schematic diagrams of four main methods of additive manufacturing: (**a**) fused deposition modelling; (**b**) inkjet printing; (**c**) stereolithography; and (**d**) powder bed fusion. Reproduced with permission from [14].

Three-dimensional printing (3D) is an AM manufacturing method that is based on CAD. It is widely used, and research and development are always making it better. The basic design is important for AM and 3D printing, and the process starts with the basic design of the parts to be made [6,7,8]. For acquiring basic design skills that can benefit from 3D printing, the required software and tools are employed, as is PC design software that is linked to the 3D printers [9]. After reading the file from the compiler, the 3D printer builds the object by adhering each layer to the preceding one [10,11,12]. To manufacture a component in 3D printing, layers are often printed. Instead of reading the parts as a whole, 3D printers read the components as separate two-dimensional layers [13]. Depending on the functionality required, 3D printers (Figure 1) were built to read various design files created in Standard Tessellation Language (STL).

As a practical and financially feasible manufacturing method, 3D printing has future-proofing qualities, including negligible material waste, little post-processing, and exceptionally cheap prices even for making complex products [15,16]. In addition to that, 3D printing possesses sustainable features such as reuse of plastics, recycling, and minimum or no emissions. Additionally, 3D printing can produce items with complex and well-designed geometries and light components with improved strength-to-weight ratios. As a result, this technique can be utilised to print precise medicine, complexly structured bone tissues, human organs, etc., in bioengineering or Pharmaceuticals. The use of 3D printing enables the generation of items with environmentally friendly designs.

### Techniques Used in 3D Printing

The development of AM approaches was spurred by the need to satisfy the demand for the creation of complex models at high resolutions. A substantial amount of progress has been made in AM technologies as a direct consequence of fast prototyping. The three main types of additive manufacturing methods are sintering, melting, and stereolithography. Sintering raises the temperature of the material without liquifying it and creates complex and high-resolution prototypes. Electron beams are used to heat the particles during the melting process [17,18]. A UV laser is a kind of laser that is used for photopolymerization. The highest temperatures possible are achieved by firing a UV laser over a vat of photopolymer resin, creating a torque-resistant ceramic component. The American Society for Testing and Materials (ASTM) has divided AM into seven distinct methods, including vat photopolymerization, powder bed fusion, binder jetting, material jetting, direct energy deposition, material extrusion, and sheet lamination [19,20]. In the following sections, significant methods are described with a focus on their labour needs, the kinds of materials they use, potential applications for 3D printing, and their advantages and disadvantages [21,22,23].

The 3D printing method in the pharmaceutical industry involves important techniques such as selective layer sintering, powder bed fusion, inkjet technology, and Direct energy deposition. The brief outline of the techniques is as follows: The term “SLS” (Selective Layer sintering) refers to the earliest 3D printing method that has ever been commercialised. The first 3D printers were stereolithographic printers, which generated 3D prototypes, 3D models, 3D components, and 3D patterns. These printers also existed in the early 2000s. Fused deposition modelling (FDM) is a technique for fabricating three-dimensional objects by extruding successive layers of molten thermoplastic filament. FDM 3D printers have a support base that is constructed in such a manner that it can move vertically up and down. Due to this, they have a certain amount of leeway. The filament is heated until it reaches its critical point, and then the item is produced by a second extruder that is coupled to the bottom plate. This produces the object in a layer-by-layer fashion through a nozzle [24,25,26]. In addition to FDM, there are some other 3D printing technologies available that have potential applications in the medicinal industry, including powder bed fusion (PBF) [27,28,29,30,31,32,33], inkjet technology [12,34,35,36], and direct energy deposition (DED) [37,38,39,40,41,42,43].

In comparison to other methods, SLS technology offers several advantages, including a wide variety of raw materials, high component complexity, quick manufacturing, low production costs, and a high rate of resource use. It has become one of the most cutting-edge methods of 3D printing and has found significant use in the fields of industry, medicine, aerospace, and aviation.

More recently, Goyanes et al. [44] have published an article that provides only an overview of the role of advanced production of precise medicine and the future scope for the manufacturing methods used for precision medicine; this present work focuses specifically on the use of SLS in the manufacture of pharmaceuticals. This article provides a deep discussion on the special capabilities of SLS, such as the capacity to control the porosity of printed materials and produce amorphous solid dispersions, which are difficult to do with conventional techniques. It also examines the potential of SLS in the manufacturing of specialised dosage forms, such as porous pills that quickly dissolve when taken orally. The efficiency of different 3D printing methods for use in tissue engineering applications is also thoroughly compared in our study. This study adds knowledge by concentrating explicitly on the developments and uses of SLS in pharmaceutical manufacturing and offers new information and viewpoints for academics and industry professionals.

## 2. Three-Dimensional (3D) Printing Approach

### 2.1. Design, Working Mechanism, and Materials of Selective Laser Sintering

#### 2.1.1. Working Mechanism

**Figure 2:**
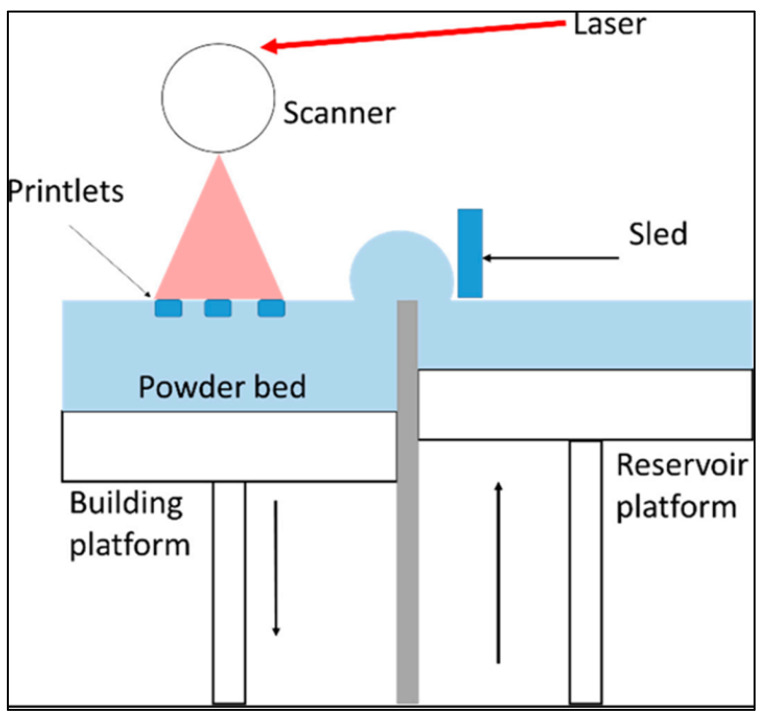
Scheme of the SLS printer. Reproduced with permission from [48].

A typical SLS arrangement is shown in Figure 2. In this case, a laser is utilised to sinter small polymer powder particles. To guarantee strong construction, the whole cross-section of the component is scanned in SLS [45,46,47]. First, the construction site and the powder bin are heated to a temperature that is only slightly below the polymer’s melting point. When a re-coating blade is first constructed, the construction platform is covered with a very thin layer of powder. The particles of polymer powder are then selectively sintered, which means they are fused together using a CO_2_ laser. The process is repeated while the contour of the succeeding layer is scanned. After the application of each layer’s finishing touches, the building platform will lower, and the blade will once again paint the surface. This procedure was repeated until the piece had completely formed by the time it was finished. After the printing process, the parts are entirely covered with the powder that has not yet been sintered. The powder container must be given some time to cool down before the pieces can be unpacked. It is possible that the cooling process will take up to 12 h. After that, the SLS-printed items are prepared for use, either with or without a further post-cleaning operation, and this may be accomplished by blasting or cleaning with compressed air.

#### 2.1.2. Materials

a. Powders

**Figure 3:**
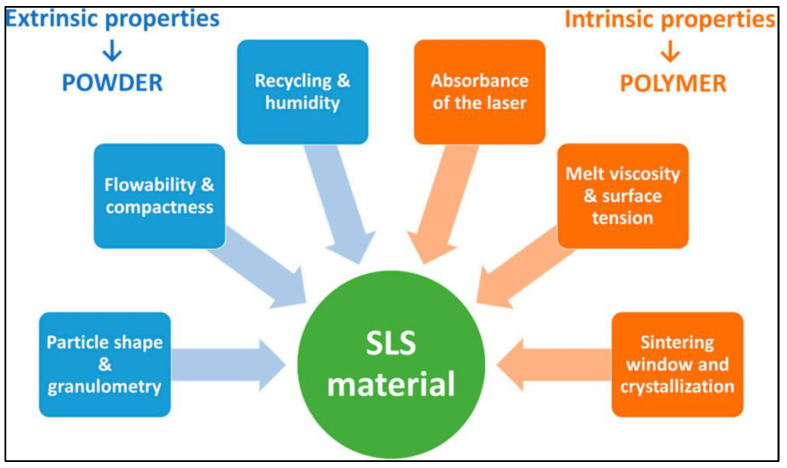
The most important criteria for polymeric powder printability in SLS.

The parameters for the selection of materials for SLS are shown in Figure 3. SLS may be used to treat almost any form of polymeric material, provided that the material in question has a powder particle with a tendency to sinter or fuse when heated [49,50,51,52,53]. By making use of a sacrificial binder material (which is often a polymer), matrix powders may be fused together by the process of laser sintering [54,55,56]. After the whole part has been sintered, the sacrificial binder might be removed by de-bonding the “green” component in a thermal furnace. This would be performed after the complete part has been sintered. By using a sacrificial binder, one is able to broaden the variety of materials that may be laser-sintered [57,58,59,60]. However, as compared to other methods of rapid prototyping, the range of materials (powders) that may be laser sintered without the need for a sacrificial binder is very extensive [61,62,63]. In this procedure, a laser is used as the power source to sinter granulated material (typically polyamide or nylon). The laser is automatically pointed at certain locations in space in a 3D model, connecting the material to produce a sturdy structure [64,65]. Although they are carried out under distinct technical conditions, the functions are comparable to selective laser melting (SLM). When employing this laser on powders with lower melting or sintering temperatures, however, a liquid binder is often used. SLS is used in conjunction with a range of polymers, alloys, and metal powders, even though SLM is often appropriate for particular metals, such as steel and aluminium [66,67,68].

b. Thermo plastics

Nylon, a common thermoplastic used in engineering that is well-known for its lightweight properties as well as its strength and durability, has been extensively employed as the material that has been used most commonly by early SLS 3D printers [69,70]. Due to its exceptional durability, nylon is an excellent material option for both rapid prototyping and large production [71,72,73]. Nylon is still the SLS material that is used the most often nowadays. The technological progress that is now being seen in 3D printing has contributed to the creation of nylon composites filled with carbon, glass, and aluminium fibres. These composites have enhanced mechanical and thermal properties. Poly(ether ether ketone) (PEEK), thermoplastic poly(urethanes), poly(methyl methacrylate), and poly may all be processed by contemporary SLS 3D printers in addition to nylon (carbonate) [74,75,76]. The last two polymers both have properties that make them resistant to sparks and static electricity.

c. Amorphous polymers

Products that have great dimensional accuracy, feature resolution, and surface quality may also be manufactured using amorphous polymers with varied grain sizes, such as poly(carbonate). These types of polymers can also be employed. Since amorphous materials have not been used in the production of components that need great strength and endurance, it is not necessary for them to be completely solidified. Casting epoxy moulds is one of the applications for SLS masters, along with the production of silicone rubber [77,78].

d. Metals

SLS is one of the few techniques of rapid prototyping that permits the direct manufacture of metallic goods without the need for a polymer fastener. This makes it one of the most advantageous features of SLS. CAM-LOM, which stands for Computer Aided Manufacturing-Laminated Object Manufacturing, is one example of a tool that is used for the direct manufacturing of metallic components. CAM-LOM monitors 3D laser cladding cycles [79,80,81]. These elective cycles, however, are often accompanied by processing in order to mitigate the adverse effects of arousal that are brought on by the lack of predictability and exactness in the environment (perhaps on a single machine) [82,83,84,85]. Table 1 shows the summary of the materials used, dimensions of the printlets, and drug release profiles in the reported SLS studies.

## 3. Applications

### 3.1. Biomedical Engineering Applications of SLS

In the field of biomedical engineering, SLS may be used for a variety of different purposes. In the sections that followed, an examination of the applications of biomedical technology was conducted, with a particular emphasis placed on drug and biomolecule delivery systems as well as scaffolds for tissue engineering.

a. SLS in Pharmaceuticals

The SLS technology provides a platform for the construction and creation of 3D-printed medicines with almost any drug molecule in a variety of sizes, colours, textures, and flavours to enhance their appeal to various patient groups, particularly the young or the seniors, and their compliance with the treatment that is prescribed to them. The manufacturing procedure that is based on 3D printing is flexible enough to accept low as well as high medicine concentrations and allows for precise dosage strength control.

A patient-specific strategy for treating illnesses that is very precise and accurate in dosage is called personalised medicine. It is a patient-centred dose formulation that takes physiologic variables in patients—like age, weight, and present physical conditions—into account. As inappropriate dosages can be the root of 75–85% of unpleasant conditions, SLS 3D printing can help minimise the unwanted effects of conventional dosage forms. Compared to other dose forms, oral administration has benefits, with tablets being a popular type. In order to cure issues with the skin and hair, SLS 3D printing is also employed in cosmetics and nutraceuticals. Skin sensitivity is taken into account. For each patient, the appropriate level of safety and safeguards can be used. For each person, nutritional supplements are provided via nutraceuticals. Dental applications, including oral and maxillofacial surgery, can benefit from 3D printing [86,87].

**Figure 4:**
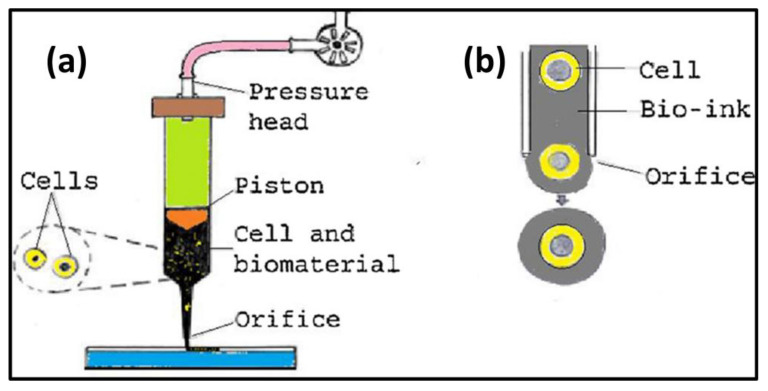
Pictorial representation of a bio-printer (**a**,**b**) a printer head. Reproduced with permission from [89].

**Figure 5:**
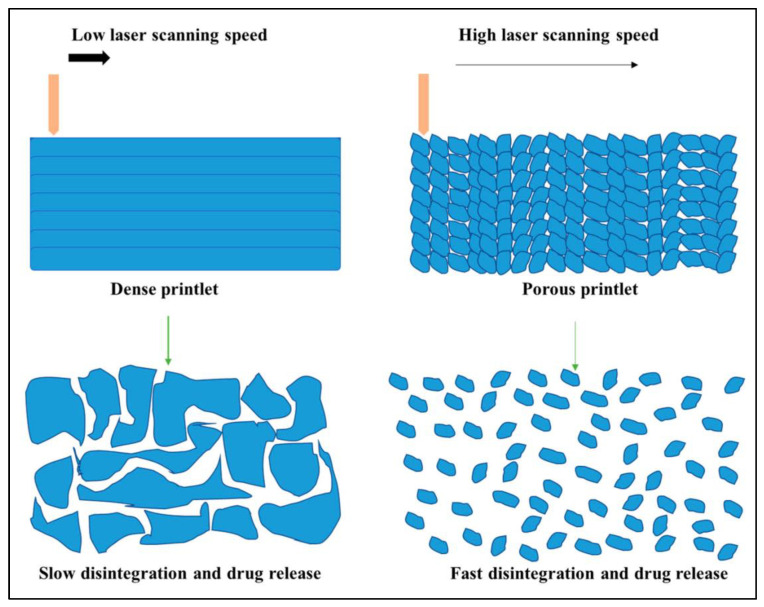
Relationship between laser scanning speed, porosity of the printlets, and disintegration.

Figure 4 and Figure 5 show how pharmaceuticals are typically loaded into three-dimensional composites either by mixing them with polymer powders before subjecting them to SLS or by adsorbing them onto surfaces in order to produce the composites. The poly(caprolactone) (PCL) and progesterone (PG, a steroid hormone) powder mixtures that were used to produce the PCL-PG medicine delivery system were sintered. Alternately, pharmaceuticals or biomolecules might be encapsulated in microspheres before being used in the SLS process. SLS was used to construct Ca-P/PHBV nanocomposite-based microspheres loaded with bovine serum albumin (BSA), as well as to build extremely accurate 3D scaffolds from the BSA-laden Ca-P/PHBV microspheres. This was accomplished by loading the Ca-P/PHBV nanocomposite-based microspheres with BSA [88].

**Table 1:** Summary of the materials used, dimensions of the printlets, and drug release profiles in the reported SLS studies.

Polymers	Active Ingredients	Other Components	Dimensions of the Printlets	Drug Release Profile	Reference
Kollicoat IR, Eudragit L100-55	Paracetamol	-	Cylindrical (10 mm diameter × 3.6 mm height)	Immediate and sustained	[5]
Kollidon VA64, Hydroxypropyl methylcellulose (HPMC)	Paracetamol	-	Cylindrical (10 mm diameter × 3.6 mm height)	Immediate and fast	[5]
Polyethylene oxide (PEO), Eudragit RL, Eudragit L100-55, Ethylcellulose	Paracetamol	-	Cylindrical and gyroid lattice (10 mm diameter × 3.6 mm height)	Fast, immediate and sustained	[90]
Eudragit L100-55, HPMC	Paracetamol	-	Cylindrical (10 mm diameter × 3.6 mm height), torus (10 mm diameter × 4 mm height) and square (10 mm side and 0.5 mm thickness)	N.A.	[91]
Kollicoat IR, Ethylcellulose	Paracetamol, Ibuprofen	-	Spherical (1- and 2-mm diameter)	Immediate and sustained	[92]
Kollidon VA64	Diclofenac sodium	Lactose monohydrate	Cylindrical (10 mm diameter × 3 mm height)	Immediate	[93]
Kollidon VA64	Odansetron	Mannitol, cyclodextrin	Cylindrical (12.4 mm diameter × 3.6 mm height)	Fast	[87]
Kollidon VA64	Paracetamol	-	Cylindrical (10 mm diameter × 3.6 mm height)	Fast	[87]
Kollidon VA64	Clindamycin palmitate hydrochloride	Lactose monohydrate and microcrystalline cellulose	Cylindrical (10 mm diameter × 3 mm height)	Immediate	[87]
Kollidon VA64	Ritonavir	Silicon dioxide	Cylindrical (10 mm diameter × 4 mm height and 12 mm diameter × 5 mm height)	Sustained	[94]
Kollicoat IR	Lopinavir	Lactose monohydrate, talc	Cylindrical (4.5 mm diameter × 3 mm height)	Fast and immediate	[93]
Kollicoat IR	-	-	Cylindrical (10 mm diameter × 3.6 mm height)	N.A.	[93]
Eudragit EPO, Polyvinylalcohol (PVA), Polyethylen-glycol (PEG), Carboxymethyl starch sodium, Eudragit RL, Stearic acid, HPMC, Ethylcellulose, Kollicoat MAE	Indomethacin, Nifedipine, Astragalus polysaccharin, Ibuprofen, Metoprolol, Tinidazole, Paracetamol, Diclofenac sodium, Bletilla striata	-	2D structures (0.5 mm height): circle (10 mm diameter), triangle (12 mm side), honeycomb (10 mm diameter), moon (10 mm length), star (14 mm length), number 1 (10 mm length); and their respective 3D printlets (4 mm height)	Sustained and immediate	[48]

#### 3.1.1. Active Pharmaceutical Ingredients (API)

Active components were used as tracers in a significant amount of the research that was carried out to determine how the SLS process influenced the drug’s release, physical condition, and thermal degradation. In spite of the fact that active product ingredients often constitute a smaller portion of the polymeric matrix, Yang et al. have recently constructed 2D structures that merely contain API without the need for a polymeric carrier. SLS is more tolerant of low polymer loading compared to fused deposition modelling (FDM), in which filaments manufactured with 30% less polymer could not be used for printing owing to their brittleness. In SLS, however, printing is possible with filaments generated with low polymer loading [95,96,97]. It is fascinating to see that 3D modelling has been used in the past to investigate the sintering of application programme interfaces [88,98,99]. These studies have contributed to the demonstration that a number of application programme interfaces, including progesterone and ibuprofen, absorb light at the CO_2_ laser beam because carbonyl groups are present, which intensifies the sintering process. In detail, due to the instrument’s huge print volume and high packing density, the SLS approach may provide certain advantages over other 3D printing techniques to produce larger quantities of dosage forms (such as 30 or 100 tablets per print). The process uses a laser beam of a certain wavelength as a source of power to selectively fuse powder particles on the surface of a powder bed. The kind of laser can range from laser diodes to CO_2_ lasers, depending on the needed power and optical characteristics of the original powder formulation.

#### 3.1.2. Fillers and Additional Ingredients

In addition to thermoplastic polymers, active chemicals, and absorbance enhancers, more intricate formulations that include additional substances have also been researched. For example, cyclodextrin complexes may be used as a dissolving enhancer; mannitol can be used as a taste masker; lactose can be used as a filler; and microcrystalline cellulose (MCC) can be used as both a filler and a binder [100,101,102]. It is essential to take into consideration the fact that none of the following excipients had any impact on the sintering process. In addition, difficulties with spread ability have been handled thanks to the use of flow boosters such as silicon dioxide and talc. This is especially true for drugs that may limit the powder’s capacity to flow when given in large dosages. Although the use of multi-component mixtures may improve some printlet characteristics, it also carries the risk of causing damage if the components interact with one another [103,104].

#### 3.1.3. Orally Disintegrating Printlets (ODPs)

One of the most advantageous features of this technology is its porosity, which makes it possible to create ODPs with individualised drug release patterns. When it comes to their structural characteristics, orally dissolving forms are often not extremely tough. In addition, porosity is a natural characteristic of energy density that regulates the amount of melting that occurs in particles. This option determines how the API transforms into an amorphous state when exposed to heat. For example, increasing the scanning speed would result in a porous print layer with a rapid disintegration rate; however, this would only permit selective melting and result in poor API amorphization. Increasing the scanning speed would also result in poor API amorphization [105].

#### 3.1.4. Controlled Release Printlets

SLS employs a method for controlling medication release known as making printlets with a thick shell. This method has the potential to delay medium absorption as well as drug breakdown. The employment of this technology has allowed for the printing of three-dimensional objects that include porous microstructures and thick walls. It has been shown that a model dye may remain in the air for an extended period of time. The skywriting effect is common in SLS and occurs when an excessive amount of energy is imparted at the beginning and end of a single line of sintered material as a result of the acceleration and deceleration of the laser beam. This results in the formation of thick walls [106]. As a direct consequence of this, composite three-dimensional structures were created with a porous matrix core and a dense shell.

### 3.2. Tissue Engineering Scaffolds

Autografts, bone grafts, and other biomaterials are some of the traditional therapies for damaged or diseased tissues that may be found in the human body (natural or man-made, including biopolymers, metals, ceramics, and composites). In this aspect, substantial limitations continue to exist, including a dearth of bioactivity, the possibility of graft rejection, and a lack of donor tissue. Tissue engineering is the process of generating biological substitutes that work with the body and develop into new tissue at the location of an injury or sickness. This process may be used to treat a variety of medical conditions. In scaffold-based tissue engineering, it is essential for the clinical use of the scaffold that its macro- and micro-architectures be controlled and that the scaffold achieve a one-of-a-kind design with a complex anatomic form. This is in order for the scaffold to be put to clinical use [106]. 

Scaffolds made of biopolymers (whether or not they are biodegradable), ceramics, and composites may be fabricated with the use of SLS for the purposes of bone tissue engineering or the healing of bone tissue. A simpler SLS setup was used in order to sinter implants that were fabricated from ultrahigh molecular-weight polyethylene. Sintered materials also include PEEK and a variety of other powdery, non-biodegradable polymers. Single-component biomaterials, on the other hand, are not capable of fully satisfying the expectations for alternate bone transplantation methods. As a direct consequence of this, SLS was used in the process of fabricating composite scaffolds made up of hydroxyapatite (HA) and non-biodegradable polymers such as HA/PEEK and HA/high-density polyethylene (HDPE) [107,108,109].

It is possible to construct scaffolds out of SLS for the purpose of healing or regenerating tissues other than bone. Epoxy resin E-12 was used as a binder when K_2_O-Al_2_O_3_-SiO_2_ series dental glass-ceramic powder was combined with it by Liu et al. in order to produce fine powder composites [110]. After that, dental restoration devices using SLS that were made using these composites were created. In the process of cardiac tissue engineering, scaffolds made of PCL and SLS were used to provide support for C_2_C_12_ myoblast cells. These scaffolds have specialised mechanical and architectural properties. PCL scaffold with a 3D flow-channel network that branches and links, and then they SLS-fabricated the scaffold so that they could manufacture liver tissues that could be implanted. This allowed the researchers to create livers that could be transplanted. Table 1 shows the summary of the materials used, dimensions of the printlets, and drug release profiles in the reported SLS studies.

### 3.3. Competitors for SLS in Tissue Engineering Scaffolds

a. 3D Printing Techniques for Scaffold Fabrication

**Figure 6:**
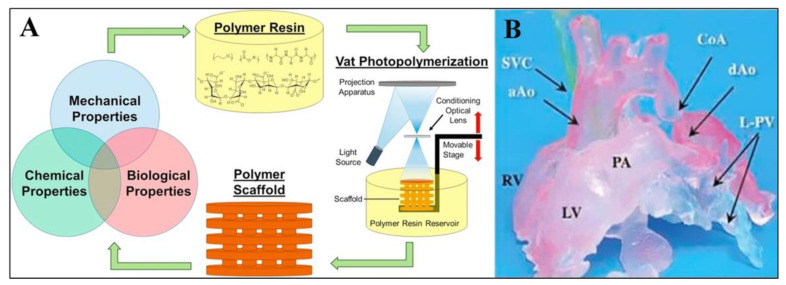
Polymer scaffold fabricated with the SLA approach. (**A**) Schematic diagram. (**B**) Products manufactured by vat-polymerization-based printing methods. Reproduced, with permission, from [111]).

In the last ten years, a variety of methods, including gas foaming, solvent casting/particulate leaching, melt moulding phase separation, electrospinning, freeze drying, and self-assembly, have been used in order to build porous three-dimensional biomimetic scaffolds. Figure 6 illustrates one example of the preparation of a scaffolding system. It is possible to employ scaffolds to recreate, on a nanoscale level, the architecture of the natural extracellular matrix. This architecture is the primary foundation for the regeneration of new tissue and serves as the basis for the process. In the beginning, the process of developing scaffolds via tissue engineering was carried out in a “top-down” fashion. During this procedure, cells are seeded onto a scaffold that is biocompatible and biodegradable. The cells then spread throughout the scaffold and fill it up, with the goal that they will eventually develop their own matrix. Using this method, it was possible to effectively produce avascular tissues such as the bladder and the skin, among other examples.

However, due to the limited diffusion capacities of these scaffolds, this method runs into a number of challenges when it is used to produce more complex tissues like the heart and liver. “Bottom-up” strategies are what have been developed in order to find a solution to this problem. Bottom-up approaches include cell aggregation via self-assembly, cell self-assembly, and cell encapsulation using microscale hydrogels. Direct cell printing is another example of a bottom-up technique [112,113,114].

b. Direct 3D printing

The term “three-dimensional printing” refers to a manufacturing process that creates three-dimensional objects by successively depositing layers of material using a computer-controlled method. In the 1990s, researchers at the Massachusetts Institute of Technology (MIT) developed the first “3D printer,” which was based on the technology of an inkjet printer at its core [115]. This printing method is also known as “binder jetting” and “drop-on powder,” both of which are names that may be used to describe it. When printing on a standard 2D inkjet printer, the ink nozzle is moved gradually from side to side along a single plane. This allows the printer to print in two dimensions: length and breadth. A similar method is used in a 3D printer, but in addition to moving left to right along a single plane, it also contains a platform that can move up and down (by an angle of 90 degrees) [116,117,118].

c. Bio-plotter Printing

**Figure 7:**
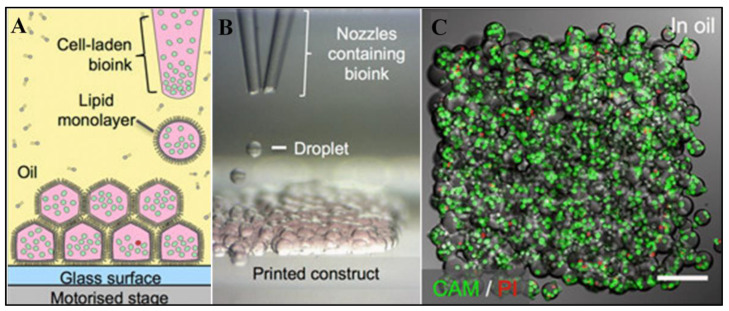
Printing with droplets in three dimensions. (**A**) Flowchart illustrating droplet-based cell printing. (**B**) Bright-field images of patterned cell connections with two different kinds of cells. (**C**) Confocal fluorescence microstructures of printed under-oil cell constructions (Scale bar = 150 μm). Reproduced, with permission, from [120].

The “bioplotter printing” rapid manufacturing technique includes the extrusion of materials via nozzles after the materials have been subjected to heat or chemical processing (Figure 7). As is the case with all methods of 3D printing, the creation of a CAD comes first, followed by its submission to the 3D printer. As the layers of the components are stacked atop one another, there is a possibility that each layer will include a combination of different kinds of materials. In order to produce the best scaffold structure possible, the Bioplotter printer has the capability to employ “bioink” and change it out in a way that is similar to how ink cartridges in an inkjet printer function. The ability of Bioplotter systems to print cell-laden gels, typically in combination with the use of other polymeric materials such as PCL, is one of the most important characteristics that leads to the production of viable and helpful scaffolds [119].

d. The role of SLS in tailoring the precise medicines

There have been several attempts to employ different additive manufacturing techniques, such as fused deposition modelling (FDM), semi-solid extrusion (SSE), direct powder extrusion (DPE), stereolithography (SLA), and SLS, for the creation of solid dosage forms. The SLS method, which enables 3D printing with pharmaceutical-grade materials, is extremely important, but its full potential and bounds have not yet been fully realised. A conventional, tightly regulated preformulation technology may be combined with a cutting-edge formulation process for the final dosage form thanks to additive manufacturing based on powder material. The earliest SLS method investigations for medicinal applications began in 2001. Today, it has been shown that it is possible to construct a broad variety of dosage forms and drug delivery systems, including implants, intrauterine devices, and quick-release or orodispersible printlets. For patients with visual impairment, it is possible to build orodispersible printlets with Braille and Moon patterns because of manufacturing flexibility. Fina et al. have described the production of flat-faced shape printlets as well as gyroids for controlled release dosage forms. Mini printlets, or pellets, have also been produced as controlled-release dose forms [121].

### 3.4. Challenges Ahead

In the field of tissue engineering, it is very necessary to exert control over the mechanical properties and degradative behaviour of sintered scaffolds. These properties may be affected in a variety of ways, including by the biodegradable materials that were used, the scaffold design, and the optimisation of the SLS parameters. The SLS approach for constructing scaffolds has a number of disadvantages, including the inability to use hydrogels and the inability to encapsulate cells inside scaffolds. Neither of these features is possible. Utilising contemporary bioreactors and the appropriate cell seeding processes should result in an equal distribution of cells over 3D scaffolds and inside the pores of the scaffolds.

Because they lack pharmacological inducers like growth hormones, the scaffolds used in tissue regeneration may not be able to promote spontaneous recovery and the restoration of functional tissue. This is likely the case because growth hormones and other pharmacological inducers encourage cell proliferation and differentiation. Even though biomolecules can be integrated into 3D scaffolds by sintering biomolecule-loaded microspheres or mixed powders of biomolecules and polymer granules, the heat generated by SLS may cause severe damage to the biomolecules. This is the case even though biomolecules can be integrated into 3D scaffolds. After the SLS manufacturing process, one potential solution to this problem is to bind biomolecules to scaffolds [122]. 

Chemotactic signals, such as growth factors, may be incorporated into and released from an organism in order to make cell homing easier. It is possible to add two distinct growth factors to surface-modified composite scaffolds that already contain immobilised heparin. Some examples of these growth factors are fibroblast growth factors, bone morphogenetic proteins, and vascular endothelial factors. It is anticipated that the growth factors would be delivered in a manner that is distinct from one another, constantly, and in a manner that is under control, therefore coordinating the activation of tissue regeneration. A significant problem persists in the absence of vascularization inside three-dimensional scaffolds designed for the regeneration of certain types of tissue [123]. 

In the field of stem cell research and application, the use of 3D printing technology presents both challenges and limitations. To begin, despite the fact that 3D printing may produce individualised and precise scaffolds, it also comes with large costs for medical and scientific research, in addition to a dearth of opportunities for mass manufacture. In addition, the production techniques of certain 3D-printed scaffold materials are both poisonous and pathogenic, which severely restricts their use in clinical settings, molecular genetics research, and the regeneration of tissue. Despite the adaptability of 3D printing technology, there are still significant obstacles to overcome in the fabrication of sophisticated geometric forms made of composite materials, in the processing of a variety of materials, and in the post-optimisation of compound surface properties. The rapid development of stem cell-based therapies for regenerative medicine has also led to an increase in the challenges presented by stem cell bioprinting in the areas of social ethics, legal logic, and government regulation [124,125].

### 3.5. Future 3D-Printed Biomaterials

The construction of effective scaffolds for the exploitation of cartilage for stem cells, extracellular bone, and matrix becomes most promising as existing technologies are further developed and more bio-ink materials come into existence. People who have organ dysfunction and abnormalities brought on by trauma or lesions may benefit from 3D-printed tissue engineering scaffolds as a method to enhance their quality of life. These people may be able to improve their condition with the use of this technology. The development of novel printing materials and nanomaterials, in particular composite materials, biocompatible materials, and complex biomaterials, will be essential to the progression of 3D printing technology in the coming years. This will be the case regardless of the specific requirements of any given application. In addition, in order to promote the standardisation, systematisation, non-toxicity, risk-freeness, environmental friendliness, and environmental conservation of 3D printing, as well as to continually increase the integration of stem cell and printing technology [126].

## 4. Conclusions and Future Scope

The SLS has emerged as a potentially valuable manufacturing technique for formulating pharmaceuticals and structural biomedical components. The advantages of SLS, such as its flexibility in handling various materials, ability to manufacture complex components quickly, and cost-effectiveness, have positioned it as a promising technology in pharmaceutical manufacturing. The focus has been on printing solid oral forms with an emphasis on personalised treatment, utilising SLS as the foundational formulation method. Notably, the SLS component of solid oral forms shows significant potential for application in orally disintegrating printlets. Despite the promising prospects of SLS as a precision medicine tool, certain technical and regulatory challenges must be addressed. Further research is needed to gain a better understanding of the printability of medicinal polymers and to optimise the process of adjusting the laser wavelength used in SLS. Due to the possibility of light-induced breakdown of organic compounds, in-depth investigations are necessary to comprehend how SLS affects the physicochemical properties and stability of pharmaceuticals. Moreover, SLS, being an AM technique, explores new possibilities for the production of multidrug or multicomponent pharmaceutical formulations capable of addressing a variety of pathobiological concerns and disorders through layer-by-layer synthesis. The ability to modulate porosity also offers control over the formulation’s stability and medication release kinetics, enhancing the overall therapeutic potential. The reviewed paper has provided valuable insights into the advancements and applications of SLS in pharmaceuticals, serving as a valuable resource for researchers and industry professionals. The future scope of SLS in pharmaceuticals and tissue engineering is promising. Researchers and manufacturers should focus on optimising the mechanical properties and degradative behaviour of sintered scaffolds, as well as finding innovative ways to incorporate hydrogels and encapsulate cells inside the structures. The preservation of biomolecules during the SLS process remains a challenge, and further advancements in this area are crucial to harnessing the full potential of SLS in drug delivery systems. Additionally, continuous research and development efforts should be directed towards exploring new 3D printing materials, nanomaterials, and complex biomaterials to enhance the standardisation, biocompatibility, and environmental friendliness of 3D-printed biomaterials.

## Data Availability

Not applicable.
